# First Characterization of Kombucha Beverages Brewed in Argentina: Flavors, Off-Flavors, and Chemical Profiles

**DOI:** 10.1155/2024/8677090

**Published:** 2024-09-09

**Authors:** Tobías Moccia, Carolina Antuña, Clara Bruzone, Julieta Amalia Burini, Diego Libkind, Lucía Paula Alvarez

**Affiliations:** Centro de Referencia en Levaduras y Tecnología Cervecera (CRELTEC), Instituto Andino Patagónico de Tecnologías Biológicas y Geoambientales (IPATEC), Consejo Nacional de Investigaciones Científicas y Técnicas (CONICET), Universidad Nacional del Comahue (UNCo) 8400, San Carlos de Bariloche, Río Negro, Argentina

**Keywords:** fermentation, fermented beverages, SCOBY, sensory analysis, sugar

## Abstract

Kombucha is an acidic beverage obtained through the fermentation of sweetened tea by bacteria and yeast. The kombucha market is relatively new and shows sustained growth. Given kombucha's inherent characteristics, compliance with regulations may be challenging. The aim of this study was to characterize the chemical and sensory diversity of commercial kombuchas in Argentina. Acetic acid was the predominant acid, while lactic and glucuronic acids were present in 70% and 50% of the kombuchas, respectively. Only 20% of the kombuchas met the ethanol limit, while all fell within the established pH range. Ninety percent had sugar concentrations exceeding the limit set by Argentinian Law. As assessed by a trained panel, drinkability was positively associated with sweetness and fruity tastes and negatively associated with astringency, bitterness, and off-flavors. This work represents the first comprehensive analysis of kombuchas in Argentina, revealing their variability and providing relevant insights for producers and consumers.

## 1. Introduction

Kombucha is a sweet, acidic, and carbonated beverage obtained by the fermentation of a tea infusion by a symbiotic consortium of bacteria and yeast (SCOBY) [1]. Kombucha, traditionally brewed at home, has gained popularity, leading to widespread industrial production [2]. This millenary beverage is composed of vitamins, polyphenols, amino acids, essential minerals, and organic acids which exert health-promoting effects such as antioxidant, detoxification and protection of liver and blood, antidiabetic, antimicrobial, reductions in blood pressure, and cholesterol [3, 4]. During the fermentation process, yeasts convert glucose into ethanol and carbon dioxide. Simultaneously, ethanol is partially oxidized by acetic acid bacteria (AAB) [5]. Additionally, other metabolites like lactic, glucuronic, gluconic, tartaric, and citric acids are generated. Kombucha can be flavored during a second fermentation by the addition of different herbs or fruits. The composition and sensory characteristics of the final product are influenced by multiple factors such as composition of microbial consortium, recipe (type of tea and sugar), time, temperature, and oxygen levels during fermentation [6, 7].

The production of this fermented beverage increases by approximately 30% year after year. The Latin America Kombucha Market was valued at USD 112.75 million in 2022 and is projected to triple by 2027 [8]. In Argentina, the market is quite new with around 15 commercial kombucha brands (own survey). Regulations worldwide are proposing measures to ensure kombucha quality. United States, Brazil, and Argentina include parameters such as pH ranging from 2.5 to 4.2, and an alcohol content equal or less than 0.5% v/v to be classified as a nonalcoholic beverage. However, kombucha was included in the Argentinian Food Code in July 2022, and most of the producers are still in the process to comply with the law and do not declare levels of alcohol on the labels. In the European Union, beverages and foods exceeding 1.2% v/v of alcohol require labeling [9]. Meeting these legal requirements when producing kombucha, especially in terms of controlling alcohol content, can be a significant challenge [10–13]. Moreover, the recent Argentinian Law on the Promotion of Healthy Eating (known as the “Front-of-Pack Labeling Law”) establishes that nonalcoholic beverages must have the claim “excess sugar” when 20% or more of its calories come from added sugars and are greater than 0.5 g/100 mL.

Regarding sensory analysis of kombucha commercial products, most of them are descriptive [6, 14, 15], and studies using trained panels are scarce [2, 16].

Since kombucha production is in full expansion and is considered a healthy drink, it is essential to know the chemical characteristics of the commercial products for sale. Thus, the aim of this study was to evaluate the physicochemical and sensory profiles, including off-flavors, of 10 commercial kombuchas produced in Argentina.

## 2. Materials and Methods

### 2.1. Sample Collection

Samples were provided by 10 producers from different regions of Argentina and included six cans or bottles of kombuchas from the same batch flavored with citrics, ginger, and/or herbs. Producers filled out a form with basic information about their production (S1). Kombuchas were transported refrigerated and processed within 3 days of receipt. Three of the samples were used for physicochemical analyses and three for sensory analyses. The kombuchas were coded to preserve confidentiality.

### 2.2. Determination of pH and Titratable Acidity (TA)

The pH of each product was measured using a Sartorius Pro Series pH meter (Sartorius, Göttingen, Germany) and a pH Electrode ST210 (Ohaus, NJ, USA). For TA, 10 mL of kombucha was decarbonated by agitation using a rotary shaker for 15 min, then titrated with 0.1 N NaOH. The end point was determined by the inflection point of phenolphthalein (pH 8.2) [17]. TA was reported as “mEq/L” using the following formula:

 $$\begin{array}{c} \text{TA}\left(\text{titratable acidity}\right)=\frac{\left(V\times C\right)}{\text{Vk}}\times 1000 \end{array}$$

where *V* is the volume of titrant (mL), *C* is the normality of titrant (0.1 mEq/mL), Vk is the volume of kombucha (10 mL), and 1000 is the factor relating milliliter to liter.

### 2.3. Determination of Organic Compounds in Kombucha

Organic compounds (acetic, lactic and glucuronic acids, glucose, fructose, and ethanol) were analyzed using a 600E High-Performance Liquid Chromatography (HPLC) (Waters, MA, USA) and a 717 plus autosampler (Waters), equipped with a 2414 refractive index detector (Waters). Kombucha samples were filtered (0.45 *μ*m filter) before injection (10 *μ*L). Chromatographic separation was achieved on a Rezex™ ROA-Organic acid −300 × 7.8 mm column, and the system was eluted isocratically with 0.005 N of H_2_SO_4_ at 0.6 mL/min. Standards of the compounds were used for identification (retention time) and quantification (external standard). Standards were prepared in ultrapure water at the following concentrations (g/100 mL): glucose and fructose: 10-5-2.5-1.25-0.25; acetic acid: 3-1.5-0.75-0.375-0.075: lactic acid: 1-0.5-0.25-0.125-0.025; and ethanol (% v/v): 1-0.5-0.25-0.125-0.025. The results were expressed as g/100 mL or % v/v. Samples were analyzed in triplicate and the data analysis was done with Empower 2 software (Waters).

### 2.4. Sensory Analysis

Sensory analysis was conducted in the Centro de Referencia en Levaduras y Tecnología Cervecera (CRELTEC, Bariloche, Argentina) by a trained sensory panel. The panel consisted of seven trained assessors. The panelists were previously informed about the type of product to be analyzed and only those who agreed participated in the evaluation. Individuals under 21 years old, pregnant women, or those with conditions that preclude alcohol consumption were not included in the study. Kombuchas were analyzed based on a set of sensory descriptors following the Kombucha Brewers International recommendations [18]. Descriptive analysis was conducted based on a five-point scale (0 = *not perceived*, 5 = *very intense*), and panelists rated the intensities of attributes related to odor (fruity, spiced, herbal, earthy, floral, acetic acid, lactic acid, and alcohol), taste (fruity, spiced, herbal, earthy, floral, acetic acid, lactic acid, alcohol, sweet, and bitter), mouthfeel (astringency and carbonation), and off-flavors (solvent, sulfurous, metallic, and dairy) [19]. The panelists were also asked to evaluate “drinkability” on a five-point scale (0 = *very low drinkability*, 5 = *very high drinkability*). Kombucha samples were stored refrigerated at 4°C until analysis. Samples were removed from the refrigerator and allowed to stand at room temperature until they reached 12°C for full perception of aroma and flavor (ASBC, Sensory Analysis–10) and were then served in transparent 40 mL plastic cups at room temperature, coded with random three-digit numbers.

### 2.5. Statistical Analyses

Physicochemical determinations were made in triplicate from three samples from the same batch and were visualized and analyzed with GraphPad Prism (v9.3.0) (Dotmatics, Boston, USA). Sensory results were statistically analyzed using R (v4.3.0) (R Foundation for Statistical Computing, Vienna, Austria) with the R Commander interface version 2.8-0 and Plugin FactoMineR. Descriptive sensory results were analyzed with multiple factorial analysis (MFA). To identify subgroups among samples, hierarchical cluster analysis (HCA) was made. Pairwise Pearson and Spearman correlations between chemical compounds and sensory attributes were calculated using GraphPad Prism (v9.3.0) (Dotmatics). To test the normality of these correlations, the Shapiro–Wilk test was done.

## 3. Results

### 3.1. pH and TA of Kombucha Products

**Figure 1:**
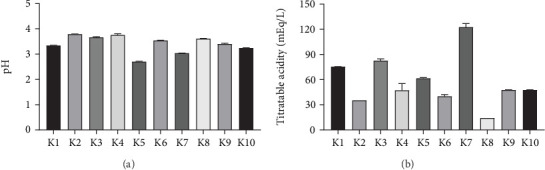
(a) pH and (b) titratable acidity of commercial kombuchas. Data are shown as the means ± SD (*n* = 3).

Kombucha products had a pH range from 2.70 to 3.80, and the TA ranged from 14.20 to 122.38 mEq/L (Figure 1). Commercial kombucha K5 had the lowest pH with 2.70 ± 0.02, while K2 and K4 had the highest pH with 3.79 ± 0.01 and 3.76 ± 0.05, respectively. Sample K7 TA was the highest with 122.38 ± 4.57 mEq/L, while K8 showed the lowest TA with 14.20 ± 0.70 mEq/L.

### 3.2. Organic Compounds of Kombucha Products

**Figure 2:**
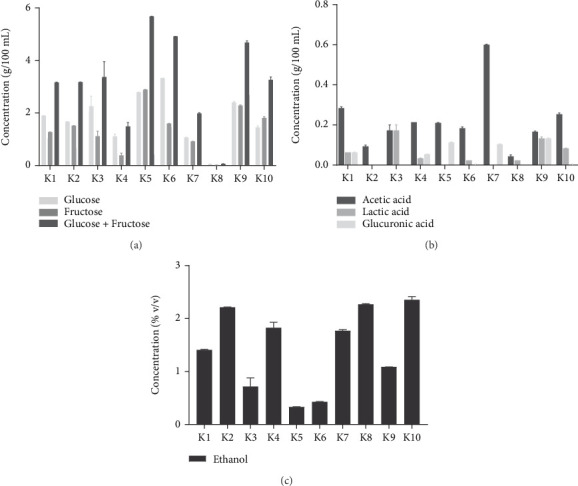
Main chemical components present in commercial kombuchas: (a) sugars, (b) organic acids, and (c) ethanol. Data are shown as the means ± SD (*n* = 3).

On average, the kombuchas showed a concentration of glucose + fructose of 3.32 ± 1.58 g/100 mL (Figure 2(a) and S1). The lowest concentration was found in K8 with 0.06 ± 0.01 *g*/100 mL, while the highest was found in K5 with 5.68 ± 0.01 *g*/100 mL.

The predominant acid in most of the kombuchas was acetic acid, with an average value for all products of 0.22 ± 0.15 *g*/100 mL (Figure 2(b)). This compound was found in all kombuchas with less than 0.30 g/100 mL, except for K7 that doubled this value (0.60 ± 0.01 *g*/100 mL). In K2 the only acid found was acetic acid and at low values (0.09 ± 0.01 *g*/100 mL). The K8 product had the lowest concentration with 0.04 ± 0.01 *g*/100 mL.

Lactic acid was detected in 70% of the products in different concentrations, with an average of 0.07 ± 0.06 g/100 mL (Figure 2(b)). K3 and K9 recorded the highest values of this acid (0.17 ± 0.03 and 0.13 ± 0.01 g/100 mL, respectively), with very similar values to those of acetic acid (0.18 ± 0.03 and 0.16 ± 0.01 g/100 mL). Lactic acid was not detected in K2, K5, and K7.

Glucuronic acid was present in 50% of the samples, with maximum levels in K5, K7, and K9 (Figure 2(b)). The average concentration of glucuronic acid in kombuchas was higher than that of lactic acid (0.09 ± 0.04 g/100 mL), but it was detected in fewer samples.

The average concentration of ethanol from all kombuchas was 1.44 ± 0.76% v/v (Figure 2(c)). K10 showed the highest concentration with 2.35 ± 0.06% v/v. The lowest concentrations were found in K5 and K6 with 0.33 ± 0.01% v/v and 0.42 ± 0.01% v/v, respectively.

### 3.3. Sensory Analysis

**Figure 3:**
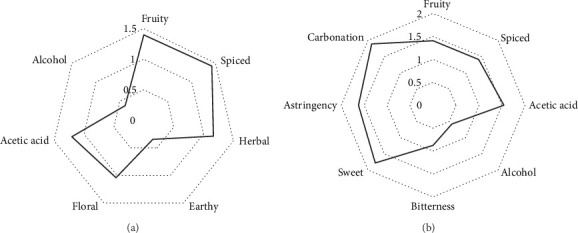
Sensory profiles of (a) odor and (b) taste in commercial kombuchas.

The sensory evaluation revealed, on average, prevalent fruity and spiced odors, with less notes of herbal and acetic odors. Floral odor was also perceived. Descriptors for alcohol and earthy odors were nearly nonidentifiable (Figure 3(a)). Regarding taste, kombuchas were described as sweet, carbonated, acidic, spiced, and fruity. A certain astringency was perceived, along with a minor bitterness, while alcohol perception was minimal (Figure 3(b)).

**Figure 4:**
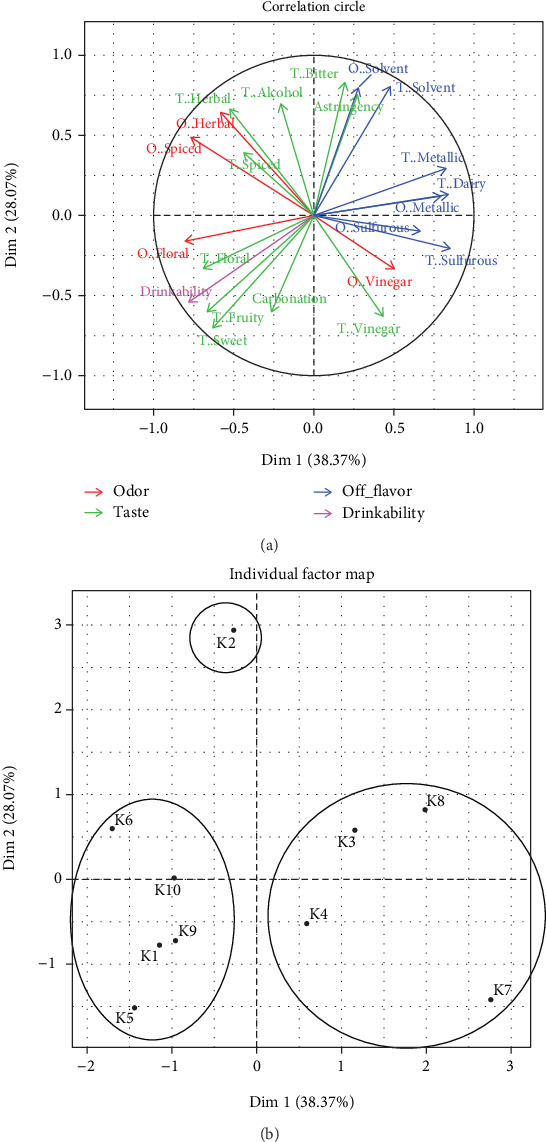
Multiple factor analysis (MFA) of sensory attributes in commercial kombuchas. O: odor; T: taste. (a) Correlation circle of sensory attributes and (b) clustering of associated samples are shown. Ellipses are drawn for three groups identified with HCA (Figure S1).

Drinkability was associated with sweet, fruity, and floral tastes and correlated negatively with off-flavors, astringency, and bitterness (Figure 4(a)). According to the HCA, the kombuchas were separated into three groups (Figure 4(b) and Figure S1). In the first group, K1, K5, and K9 showed the highest drinkability, related to sweet and fruity tastes. K6 and K10 differed from the previous ones by presenting greater spiced and herbal tastes. The second group showed higher scores in attributes not associated with drinkability. Sample K8 had high scores of off-flavors, especially sulfurous, metallic, and dairy tastes. Off-flavors were also perceived in K3, K4, and K7, although to a lesser extent. K7 was characterized by a strong vinegary taste and odor. The third group was represented by K2, characterized by herbal and spiced descriptors, while presenting an astringent, bitter, and alcoholic taste and solvent taste and odor.

### 3.4. Relationship Between Chemical Composition and Sensory Profiles

**Figure 5:**
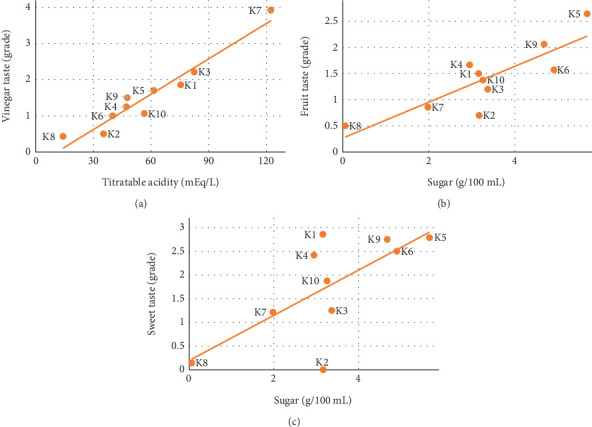
Correlations between chemical variables and sensory attributes. (a) Vinegar taste versus titratable acidity (*y* = 0.0326, *x* − 0.3584; *R*^2^ = 0.931). (b) Fruit taste versus sugar (*y* = 0.3435, *x* + 0.2655; *R*^2^ = 0.714). (c) Sweet taste versus sugar (*y* = 0.4769, *x* + 0.1949; *R*^2^ = 0.490). Other significant correlations are shown in S1. The lines are linear fit. The coding of kombuchas can be found in S1.

To assess the relationship between the chemical characteristics and the sensory profile of kombuchas, a correlation analysis was carried out (Figure 5). The concentration of acetic acid and acidity were related with the vinegary taste, indicating that the acetic acid present in the samples was perceived by the panelists (Figure 5 and S1). The fruity and sweet tastes were also perceived and were related to the amount of remaining sugars. Finally, bitter taste correlated positively with ethanol (S1).

## 4. Discussion

Kombucha is a fermented beverage whose industrial production is in full expansion [10]. Even though the market of kombucha is recent, the production of this fermented beverage has increased by approximately 30% year after year. The Latin America Kombucha Market is projected to triple by 2027 [8].

In this work, the physicochemical and sensory profile of 10 commercial kombuchas were evaluated, being the first study of these beverages in Argentina.

Regarding their physicochemical characteristics, kombuchas are characterized by a low pH and varying concentrations of organic acids, ethanol, and remaining sugars [20]. The pH of kombucha decreases as fermentation progresses due to the production of organic acids. The pH of Argentinian kombuchas was consistent with findings in other studies that reported pH ranges of 2.80–3.70, 2.90–3.40, and 3.00–3.20 in Estonian, Brazilian, and American commercial kombuchas, respectively [2, 12, 21]. According to the Argentinian Food Code, the pH should be between 2.50 and 4.20 because kombuchas with a pH below 2.50 contain substantial concentrations of organic acids, posing a potential health risk to consumers. Kombuchas with a pH exceeding 4.20 could be subject to contamination. All the kombuchas in this study met this legal requirement. Additionally, the TA of Argentinian kombuchas was variable and fell within the ranges reported by other studies [2, 12]. Tarhan Kuzu et al. [22] show that total acidity varies with fermentation time and the types of tea used in the preparations. Thus, the variability in TA could be attributed, in part, to different fermentation time and different types of tea used by producers, among other factors discussed later (S1).

The residual sugar present in a commercial kombucha depends on a large number of factors, such as the concentration of initial sugar, fermentation time and temperature, and the microbial consortium [1, 9]. The Argentinian kombuchas have shown a great variation in the concentration of residual sugars. Kim and Adhikari [10] analyzed the sugar concentrations of commercial kombuchas in the United States, finding values between 0.85 and 5.75 g/100 mL, similar to those reported in Estonian kombuchas by Andreson et al. [2]. The Argentinian Law for the Promotion of Healthy Eating states that nonalcoholic beverages must have the claim “sugar excess” when 20% or more of its calories come from added sugars and these are greater than 0.5 g/100 mL. In the case of fermented beverages, residual sugar represents 100% of the calories; therefore, the sugar limit in these drinks to avoid front labeling is 0.5 g/100 mL. In this study, only one sample was below this threshold. Thus, according to the current Front-of-Pack Labeling Law, 90% of these kombuchas should have a claim indicating “sugar excess” to alert the population of a potential health risk. However, this is contradicted by one of the last clinical trials, in which it has been proven that the consumption of kombucha reduces blood sugar and insulin levels, providing a health benefit for the consumer, even when these are sweetened [23]. It should be noted that in other countries in the region, the sugar limit to avoid front labeling is much higher (Chile: 5; Uruguay: 3; Brazil 7.5; Peru: 5 g/100 mL) (data obtained from the Front-of Package Labeling Manual of each country). Furthermore, considering that sugar-sweetened beverages have added sugar that doubles the maximum concentration found in Argentinian kombuchas, these products are still a healthier choice.

Acetic acid is the predominant acid in kombucha, and it imparts its characteristic vinegary taste [1]. This acid is produced from ethanol by AAB in aerobic conditions [24]. Acetic acid was found in all samples, and its average concentration was variable. This variability was also reported by Andreson et al. [2] in different commercial Estonian kombuchas, detecting values ranging from 0.03 to 0.64 g/100 mL. Previous studies have reported acetic acid values ranging from 0.05 to 0.35 g/100 mL [6, 14, 25–28]. However, other studies found higher concentrations, such as 1.60 g/100 mL after 21 days of fermentation [29], 1.10 g/100 mL after 30 days of fermentation [24], and 0.81 g/100 mL after fermenting for 7 days [26]. According to the data provided by Argentinian producers, fermentation time ranged from 6 to 70 days (S1) and was not related to the amounts of acetic acid found, as the kombucha with the highest acetic acid concentration fermented for 21 days, while kombuchas with the shortest and longest fermentation times had acetic acid values close to the average. Therefore, acetic acid concentration may not depend solely on fermentation time.

Lactic acid is typically produced by lactic acid bacteria from glucose. It is usually found in lower proportions than acetic acid and may not be detected. In this study, lactic acid was found in 70% of the products with values similar to those reported by Ismaiel et al. [30], and it was not detected in some kombuchas, aligning with findings in other studies [2, 31].

Glucuronic acid is considered the most important health-promoting compound in kombucha [32]. This acid acts by capturing toxins in the body, making them water-soluble and allowing their elimination through urine [33]. Glucuronic acid concentration in kombuchas is highly variable as shown in this and previous works with concentrations ranging between 0.004 and 0.186 g/100 mL [5, 6, 27].

Ethanol is one of the compounds produced by yeast and is the substrate for the synthesis of acetic acid by AAB. Ethanol concentrations measured in commercial kombuchas vary significantly, ranging from 0 to 3.33% v/v [2, 9, 12, 21]. Consistent with these values, the Argentinian kombuchas showed highly variable alcohol levels. It should be noted that only two products had ethanol levels below 0.5% v/v. The allowed levels of ethanol in nonalcoholic beverages differ between countries. While in Argentina and the United States, the limit is 0.5% v/v, some regions in Europe set a limit of 1.2% v/v. In Colombia, the maximum allowed alcohol limit for nonalcoholic beverages is 2.5% v/v [9]. Ethanol concentration primarily varies depending on the fermentation time, oxygenation, and microbial consortium [9, 34]. While yeast is almost exclusively responsible for ethanol production, the AAB transforms it into acetic acid. This process, in turn, depends on the oxygen concentration during fermentation, as AAB transforms ethanol into acetic acid in the presence of oxygen. Contact with oxygen can occur through artificial aeration during the process or through contact via the fermenter's surface, which varies depending on its geometry and exposed surface [7]. Additionally, some producers perform a second fermentation which is usually carried out in the absence of oxygen and could increase ethanol levels. It is important to note that the different types of tea and flavors added to Argentinian kombuchas (S1) could affect nitrogen levels in the process [35, 36]. Nitrogen, in turn, may impact on microbial metabolism and the amount of alcohol found in the final product [36]. Based on all these factors, controlling alcohol concentration in the kombucha production process is a challenge for producers.

Altogether, the variability of TA, acids, and ethanol in kombucha can be attributed to several factors including oxygen exposure, the specific tea blend [22, 27], initial sugar concentration, the type of sugar used [20], and any additional flavors used in the preparation [36]. Additionally, fermentation temperature and time play crucial roles as they influence the metabolic activities of bacteria and yeast, thereby affecting the metabolites they produce [37]. Another significant factor to consider is the microbial consortium itself; variations in the composition of AAB, lactic acid bacteria, and yeast species can lead to variability of fermentation products, specially ethanol, acetic, glucuronic, and lactic acid [38].

Regarding sensory profile, kombucha has been described as similar to cider, with a strong and unique taste [1, 31]. It is worth highlighting that this is the first study evaluating sensory profiles by a trained panel and the first one evaluating off-flavors in commercial kombuchas. The Argentinian kombuchas were perceived as fruity, spiced, with a vinegar odor, and to a lesser extent, with an herbal odor. Predominant odors generally included cider-like, fruity, and vinegar notes [1, 16]. Regarding taste and mouthfeel, the kombuchas were perceived as sweet, carbonated, slightly acidic, astringent, and fruity. All of this aligns with Ivanišová et al. [8], describing kombuchas as sweet-sour, vinegar-flavored, with a fruity after-taste, and pleasant to drink. Regarding the perception of chemical compounds, Andreson et al. [2] showed a positive correlation between the vinegary and floral tastes and the acetic acid and sugar values, respectively. Our results were in concordance with this study. Moreover, Andreson et al. [2] classified kombuchas into two flavored clusters: one with kombuchas that presented a sweet and fruity taste and the other with a tea or herbal taste. The kombuchas analyzed here were classified in the same way, but adding a third group with off-flavors, bitter, and astringent tastes. In this study, sulfurous, solvent, dairy, and metallic tastes negatively affect the drinkability of kombuchas when present. Thus, Argentinian kombuchas were clustered into three groups: the first group (K1, K5, K6, K9, and K10) were perceived as sweet and fruity and had high drinkability and low levels of off-flavors. In addition, K5, K6, and K9 had the highest levels of residual sugars, and K5 and K6 had the lowest levels of ethanol. K10 had the highest ethanol levels that were not perceived by the panelists, which may be explained by its high flavoring. The second group consisted of kombuchas with low drinkability, which also had the highest levels of off-flavors. The third group corresponds to an alcoholic kombucha with low drinkability which was characterized by herbal and spice descriptors, while presenting an astringent, bitter, and alcoholic taste and solvent taste and odor. It is noteworthy that most kombuchas with high drinkability had the highest concentrations of residual sugars, moderate concentrations of acetic acid, and the lowest perception of off-flavors. In Latin America, the nonalcoholic beverage market is dominated by sugar-sweetened beverages, so it is expected that sugar is perceived positively by some consumers. However, other consumer groups seek low-sugar beverages, prioritizing health. Therefore, the variability of kombuchas produced in Argentina in terms of sweetness and acidity would appeal to a broad spectrum of consumers.

## 5. Conclusions

The heterogeneity found in Argentinian commercial kombuchas is noteworthy. Acetic acid was the predominant acid, contributing to the acidity and the characteristic odor and tangy taste of kombucha. pH values were within the limits established by the Argentinian Food Code; however, in most cases, the ethanol limit was exceeded, and sugar concentrations surpassed the limit set by the Argentinian Front-of-Pack Labeling Law, posing significant challenges for producers. The sensory evaluation highlights fruity and spiced notes, with varying levels of off-flavors impacting drinkability. The findings underscore the complexity and variability of Argentinian kombuchas, providing valuable insights for producers and consumers. Moreover, the study sheds light on the potential of kombuchas with diverse sweetness and acidity levels to cater to different consumer preferences, particularly in Latin America's sugar-dominated beverage market.

## Data Availability

The datasets used and/or analyzed during the current study are available from the corresponding author on reasonable request.

## Supporting Information

Supporting Information Additional supporting information can be found online in the Supporting Information section. S1: Table 1: details of kombucha products. Table 2: physicochemical parameters of 10 different kombucha brands. Table 3: Pearson's correlation matrix for physicochemical and sensory variables. Figure 1: dendrogram based on hierarchical cluster analysis (HCA) using sensory attributes.
