# Supplementary material for: First Characterization of Kombucha Beverages Brewed in Argentina: Flavors, Off-Flavors, and Chemical Profiles
Source: Int J Food Sci. 2024 Sep 9;2024:8677090. doi: 10.1155/2024/8677090 (PMC11832255; doi:10.1155/2024/8677090)
Supplement: Supporting Information — Additional supporting information can be found online in the Supporting Information section. S1: Table 1: details of kombucha products. Table 2: physicochemical parameters of 10 different kombucha brands. Table 3: Pearson's correlation matrix for physicochemical and sensory variables. Figure 1: dendrogram based on hierarchical cluster analysis (HCA) using sensory attributes. [file 8677090.f1.docx]

**Supplementary material**

International Journal of Food Science

**First characterization of kombucha beverages brewed in Argentina: Flavors, off-flavors and chemical profiles**

## Tobías MOCCIA, Carolina ANTUÑA, Clara BRUZONE, Julieta Amalia BURINI, Diego LIBKIND, Lucía Paula ALVAREZ*

*Corresponding author: Alvarez Lucía Paula. Centro Regional Universitario Bariloche, Universidad Nacional del Comahue. [alvarez.lucia@comahue-conicet.gob.ar](mailto:alvarez.lucia@comahue-conicet.gob.ar)

**Supplementary Tables and Figures**

Supplementary Table S1. Details of kombucha products.

| Code | Origin | Container | Sugar | Tea | Flavor | Best before | Fermentation time (days) |
| --- | --- | --- | --- | --- | --- | --- | --- |
| K1 | Patagonia | Can | Organic brown sugar | Green, red and black tea | Citric, ginger and herbs | 18.07.2022 | 14 |
| K2 | Buenos Aires | Can | Organic brown sugar | Green tea | Ginger and herbs | 20.12.2023 | 20 |
| K3 | Buenos Aires | Can | Organic brown sugar | Yerba mate red and green tea | Citrics and ginger | 27.11.2022 | 21 |
| K4 | Buenos Aires | Can | Organic brown sugar | Black and green tea | Citrics, ginger and herbs | 03.09.2022 | 29 |
| K5 | Córdoba | Plastic bottle | Organic brown sugar | Green tea | Citrics and herbs | 03.03.2023 | 6 |
| K6 | Patagonia | Can | Organic brown sugar | Green tea and yerba mate | Citrics, ginger and herbs | 21.09.2022 | 21 |
| K7 | Buenos Aires | Plastic bottle | Organic brown sugar | Black tea | Ginger and citrics | 04.05.2023 | 21 |
| K8 | Buenos Aires | Plastic bottle | Organic brown sugar | Red tea | Ginger and citrics | 04.02.2023 | 28 |
| K9 | Buenos Aires | Can | Organic brown sugar | Black tea | Ginger and citrics | 10.11.2022 | 12 |
| K10 | Córdoba | Plastic bottle | Organic brown sugar | Green tea | Citrics and herbs | 05.03.2023 | 70 |

Supplementary Table S2. Physicochemical parameters of ten different kombucha brands.

| Sample | Titratable  acidity  (mEq/L) | pH | Glucose + fructose (g/100 mL) | Acetic acid (g/100 mL) | Lactic acid (g/100 mL) | Glucuronic acid (g/100 mL) | Ethanol (%v/v) |
| --- | --- | --- | --- | --- | --- | --- | --- |
| K1 | 75.47±0.01 | 3.34±0.01 | 3.16±0.01 | 0.28±0.01 | 0.06±0.01 | 0.06±0.01 | 1.4±0.01 |
| K2 | 35.32±0.01 | 3.79±0.01 | 3.17±0.01 | 0.09±0.01 | 0 | 0 | 2.19±0.01 |
| K3 | 82.53±2.16 | 3.66±0.03 | 3.37±0.58 | 0.18±0.03 | 0.17±0.03 | 0 | 0.71±0.16 |
| K4 | 47.19±8.03 | 3.76±0.05 | 2.95±0.15 | 0.21±0.01 | 0.03±0 | 0.05±0.01 | 1.81±0.10 |
| K5 | 61.52±0.88 | 2.7±0.02 | 5.68±0.01 | 0.231±0.01 | 0 | 0.11±0.01 | 0.33±0.01 |
| K6 | 40.03±2.21 | 3.54±0.01 | 4.91±0.01 | 0.18±0.01 | 0.02±0.005 | 0 | 0.42±0.01 |
| K7 | 122.38±4.57 | 3.04±0.01 | 1.98±0.03 | 0.6±0.01 | 0 | 0.1±0.01 | 1.76±0.02 |
| K8 | 14.2±0.01 | 3.61±0.01 | 0.06±0.005 | 0.04±0.005 | 0.02±0.005 | 0 | 2.28±0.01 |
| K9 | 47.64±0.33 | 3.4±0.02 | 4.68±0.06 | 0.16±0.01 | 0.13±0.01 | 0.13±0.01 | 1.07±0.01 |
| K10 | 56.54±1.21 | 3.24±0.01 | 3.26±0.10 | 0.25±0.01 | 0.08±0.005 | 0 | 2.35±0.06 |

Supplementary Table S3. Pearson correlation matrix for physicochemical and sensory variables.

|  | Titratable acidity (mEq/L) | Sugar | Acetic acid | Lactic acid | Glucuronic acid | Ethanol | pH |
| --- | --- | --- | --- | --- | --- | --- | --- |
| O. Floral |  | 0.6528 |  |  |  |  |  |
| T. Fruity |  | 0.8449 |  |  |  | -0.6842 |  |
| T. Spiced |  |  |  |  |  |  |  |
| T. Herbal |  |  |  |  |  |  |  |
| T. Floral |  |  |  |  |  |  |  |
| T. Vinegar | 0.9649 |  | 0.9088 |  |  |  |  |
| T. Lactic acid | 0.751 |  |  |  |  |  |  |
| T. Alcohol |  |  |  |  |  |  |  |
| T. Sweet |  | 0.6992 |  |  |  |  |  |
| T. Bitter |  |  |  |  |  | 0.7301 |  |
| Astringency |  |  |  |  |  |  |  |
| O. Solvent |  |  |  |  | -0.7123 |  | 0.7418 |
| T. Solvent |  |  |  |  | -0.7136 |  | 0.7137 |
| O. Metallic |  |  |  |  |  |  |  |
| T. Metallic |  | -0.8744 |  |  |  |  |  |
| O. Sulfurous |  |  |  |  |  |  |  |
| T. Sulfurous |  | -0.7276 |  |  |  |  |  |
| T. Dairy |  | -0.6383 |  |  |  |  |  |

O: Odor, T: Taste

Only statistically significant (ɑ = 0.05) coefficients are shown.


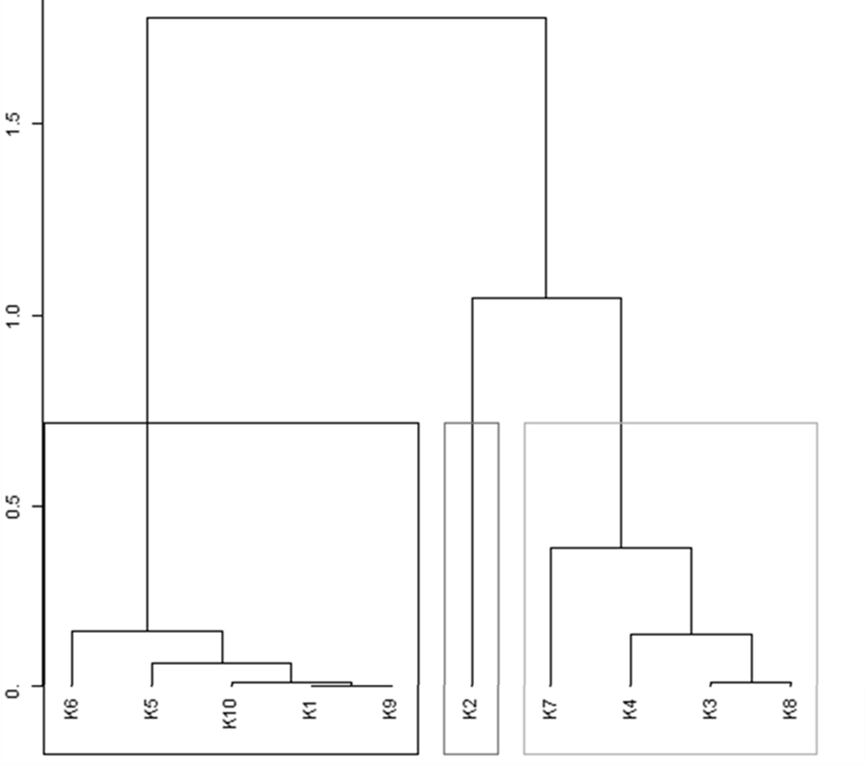


**Supplementary Figure S1**. Dendrogram based on Hierarchical Cluster Analysis (HCA) using sensory attributes
